# Changes in ankle joint motion after Supramalleolar osteotomy: a cadaveric model

**DOI:** 10.1186/s12891-017-1749-0

**Published:** 2017-09-09

**Authors:** Hak Jun Kim, Eui Dong Yeo, Im Joo Rhyu, Soon-Hyuck Lee, Yeon Soo Lee, Young Koo Lee

**Affiliations:** 1 Department of Orthopedic Surgery, Guro Hospital, Korea University College of Medicine, 80 Gurodong, Gurogu, Seoul 152-703 South Korea; 2 Department of Orthopedic Surgery, Veterans Health Service Medical Center, 53, Jinhwangdo-ro 61-gil, Gangdong-gu, Seoul 134-791 South Korea; 3 Department of Anatomy, Korea University College of Medicine University, 73 Inchon-ro, Seongbuk-gu, Seoul South Korea; 4 Department of Orthopaedic Surgery, Anam Hospital, Korea University College of Medicine, 73 Inchon-ro, Seongbuk-gu, Seoul 136-705 South Korea; 5 Department of Biomedical Engineering, College of Medical Science, Catholic University of Daegu, 330, Geumrak, Hayang-eup, Gyeongsan-si, Gyeongbuk 712-702 South Korea; 6 Department of Orthopaedic Surgery,Bucheon Hospital, College of Medicine, Soonchunhyang University, 1174 Jung-1-dong, Wonmi-gu, Bucheon-si, Gyunggi-do 420-767 Republic of Korea

**Keywords:** Ankle, Osteoarthritis, Supramalleolar osteotomy

## Abstract

**Background:**

Malalignment of the ankle joint has been found after trauma, by neurological disorders, genetic predisposition and other unidentified factors, and results in asymmetrical joint loading. For a medial open wedge supramalleolar osteotomy(SMO), there are some debates as to whether concurrent fibular osteotomy should be performed. We assessed the changes in motion of ankle joint and plantar pressure after supramalleolar osteotomy without fibular osteotomy.

**Methods:**

Ten lower leg specimens below the knee were prepared from fresh-frozen human cadavers. They were harvested from five males (10 ankles)whose average age was 70 years. We assessed the motion of ankle joint as well as plantar pressure for SS(supra-syndesmotic) SMO and IS(intra-syndesmotic) SMO. After the osteotomy, each specimen was subjected to axial compression from 20 N preload to 350 N representing half-body weight. For the measurement of the motion of ankle joint, the changes in gap and point, angles in ankle joint were measured. The plantar pressure were also recorded using TekScan sensors.

**Results:**

The changes in the various gap, point, and angles movements on SS-SMO and IS-SMO showed no statistically significant differences between the two groups. Regarding the shift of plantar center of force (COF) were noted in the anterolateral direction, but not statistically significant.

**Conclusions:**

SS-SMO and IS-SMO with intact fibula showed similar biomechanical effect on the ankle joint. We propose that IS-SMO should be considered carefully for the treatment of osteoarthrosis when fibular osteotomy is not performed because lateral cortex fracture was less likely using the intrasyndesmosis plane because of soft tissue support.

## Background

Malalignment of the ankle joint can occur because trauma, neurological disorders, genetic predisposition and other unidentified factors, and result in asymmetrical joint loading [1, 2]. Asymmetric and neutral osteoarthritis can reportedly be treated with realignment surgery [3]. Similarly, both open and close wedge supramalleolar osteotomies (SMO) have been done for the treatment of malalignment of the ankle joint in adults [4]. For medial open wedge SMO, there is some debate as to whether concurrent fibular osteotomy should be performed. One biomechanical study with open wedge SMO reported that in valgus deformities suprasyndesmotic(SS)SMO with intact fibula lead to a paradox shift of the center of force(COF) and peak pressure of the ankle joint in the anteromedial direction [5]. Many reports described that the SMO shifts the weight bearing axis to the lateral aspect of the ankle joint and reduces load on the medial aspect to correct the talar tilt [6–8]. Myerson et al. reported that a greenstick osteotomy without fibular osteotomy markedly increases the stability of the cut, and the tibia can be opened with a lamina spreader to the desired amount of correction [9]. This technique creates a hinge point for opening the osteotomy, prevents overcorrection, and maintains tension on the lateral side of the cut [9]. A deeper understanding of the nature of SMO particularly in relation to malaignment of the ankle joint is required.

Lee et al. reported that the incidence of lateral cortical fracture in medial open wedge SMO was less likely to occur at the proximal one-third of the intrasyndesmosis than the suprasyndesmosis [10]. The purpose of this study was to describe the impact of SS-SMO and intra-syndesmotic(IS) SMO with intact fibula on ankle joint motion and plantar pressure and to determine which technique seems to better re-establish ankle alignment in the absence of fibular osteotomy.

## Methods

This biomechanical study was performed to know the changes in motion of ankle joint and plantar pressure after supramalleolar osteotomy without fibular osteotomy. Ten fresh-frozen human legs (5 males; average age, 70 years) were prepared by disarticulation at the knee joint.. A normal range of ankle joint movement was established clinically.

### Specimen prepare

**Fig. 1:**
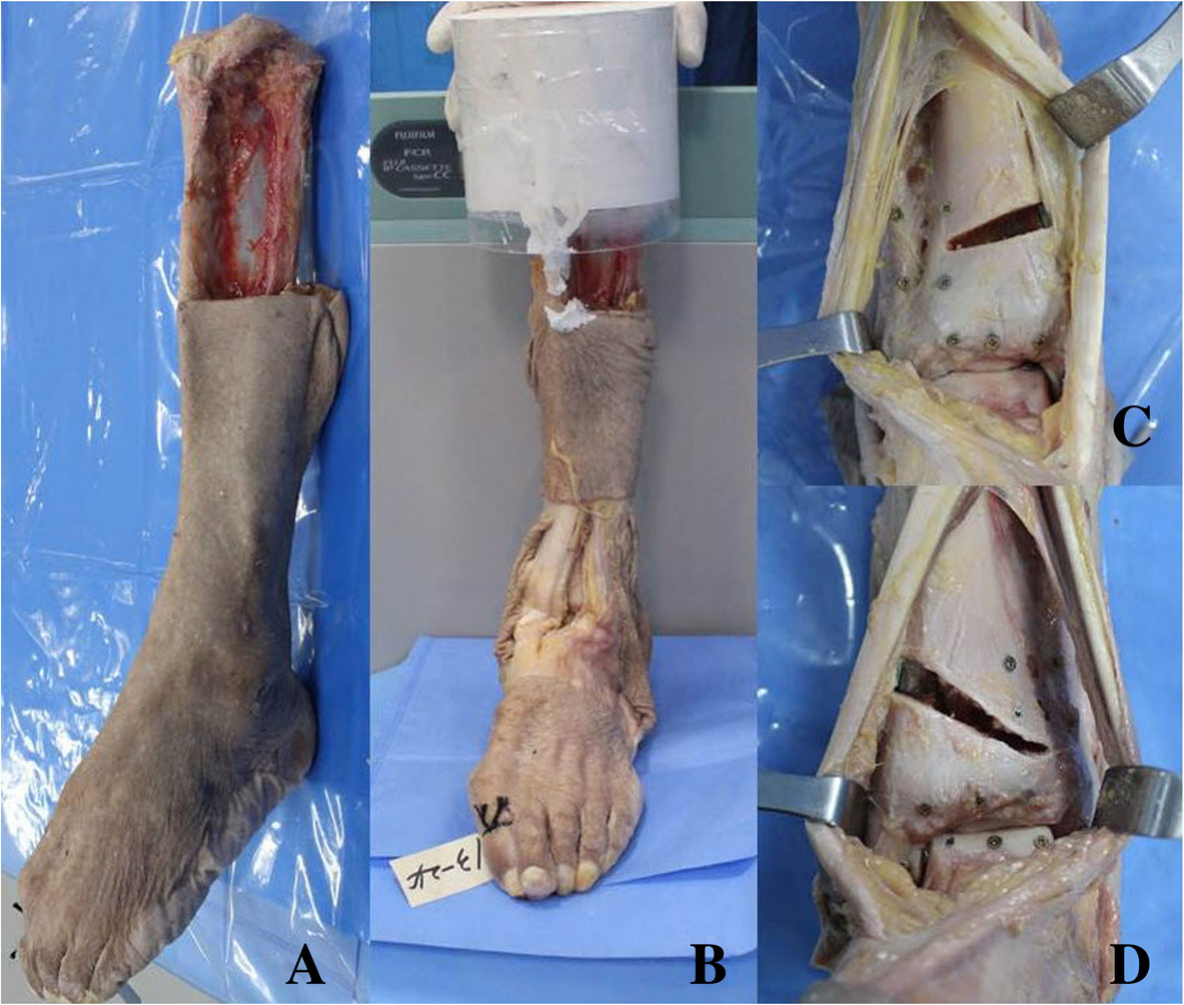
**a** lower leg preserved syndesmotic structure and tendon, ligament. **b** PMMA(poly-methylmethacrylate,zimmer) block of cylindrical shape to apply the axial load on Instron model. **c** Intrasyndesmotic supramalleolar osteotomy with wedge plate. **d** Suprasyndesmotic supramalleolar osteotomy with wedge plate

The skin and subcutaneous tissues around the knee and ankle were removed while preserving the syndesmotic structure, tendons, and ligaments (Fig. 1a). A cylindrical poly-methylmethacrylate(zimmer, Inc.; Warsaw, IN) block was used at the proximal tibia and fibula to apply axial load(Fig. 1b). For the SMO, angular deformities were created in the distal tibia. We assessed ankle joint motion after SS-SMO and IS- SMO. SS-SMO was performed on five leg (right, 3; left, 2)by using an oscillating saw in an oblique direction, mediolaterally above a line perpendicular to the tibial cortex, such that it perforated the lateral cortex 5 mm above the distal tibiofibular joint (Fig. 1c). Wedge plates(Arthrex, Inc.; Naples, FL Low profile plate, wedge size 8 mm) were used to create valgus position. IS-SMO was performed on five legs (right, 2; left, 3) such that it perforated the lateral cortex bisection point of the distal tibiofibular joint (Fig. 1d).

### Mechanical system

**Fig. 2:**
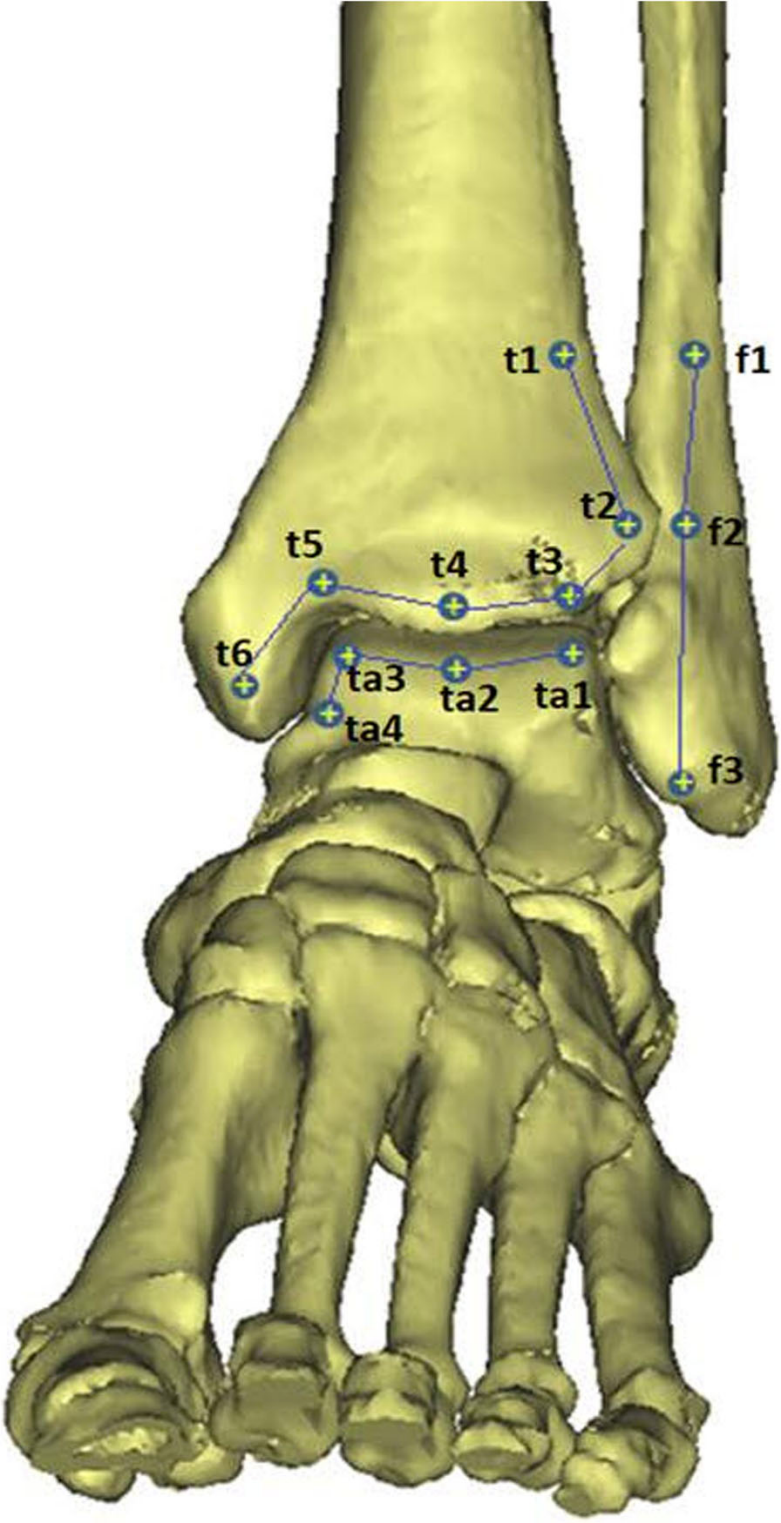
Landmarks are anchored by inserting micro cross-head screws (6 on the tibia, 3 on the fibula, and 4 on the talus). The landmarks are placed alongside joint articulation surfaces

**Fig. 3:**
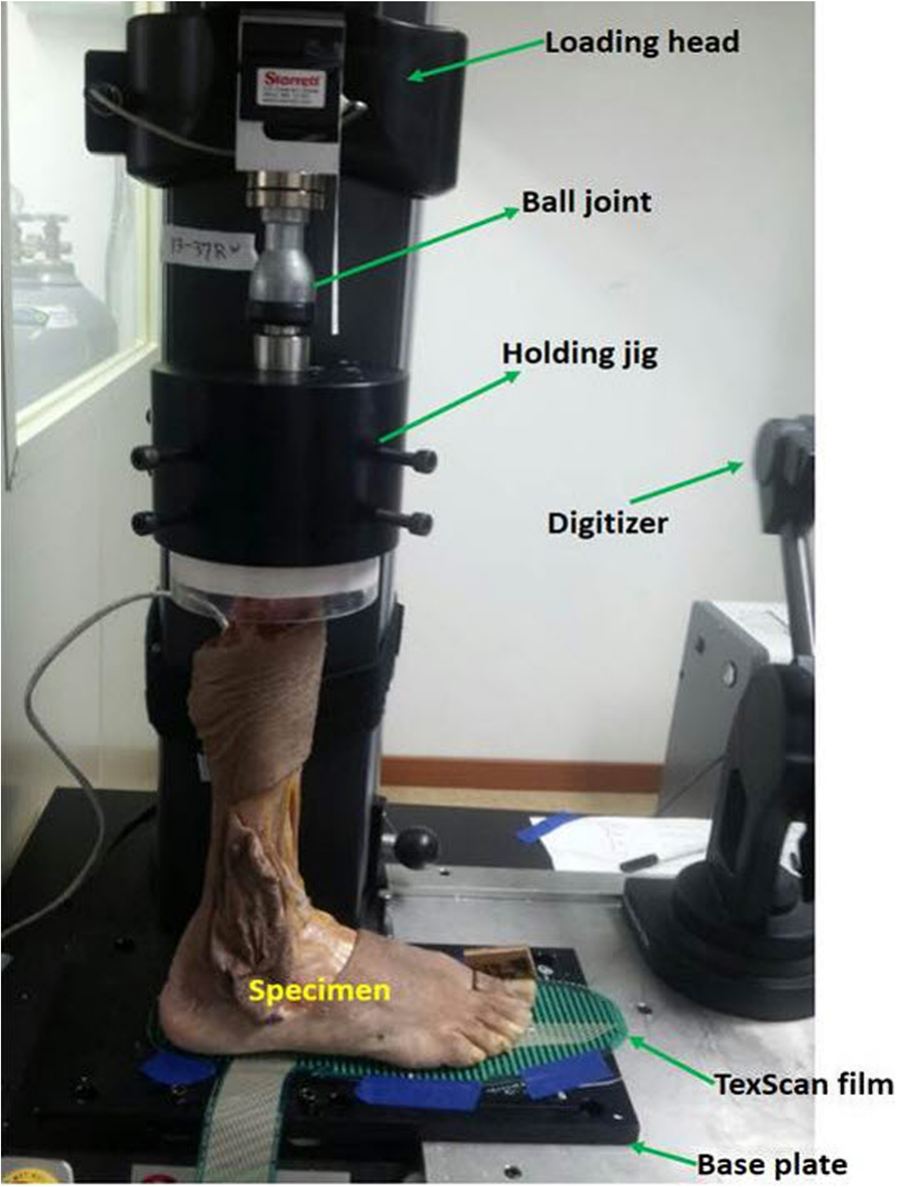
Experimental configuration. A cadaveric lower leg specimen is subjected to a compression of a single leg standing posture. The specimen connected to the loading head, through a ball joint which does not constrain the specimen’ natural rotational reaction to the loading. Kinematic data is collected using a digitizer, while loading and displacement of the loading head, and bare foot pressure are being measured by in situ digital acquisition systems

1. Mechanical loading setup
  An ankle joint motion sensor was applied on the distal tibia, fibula, and talus (distal tibia-Tm, fibula-F, talus-Ta [Fig. 2]). Each leg was fixed into a universal mechanical testing machine (Instron model; Starrett, FMS 2500, Instron Co,USA) to simulate a single leg barefoot stance (Fig. 3). Vertical alignment of the lower limb axis was adjusted visually for the measurement. To ensure consistent placement of each specimen in consecutive measurements, three point positioning (second toe, heel, and tibia crest line)was installed. Static axial compression was increased continuously from 20 N preload to 350 N representing half-body weight. Maximum load was held for 6 s, and ankle motion and plantar pressure distribution was captured until the load was released.
2. Baseline plate coordinate system
  A baseline coordinate system is assigned on the base plate (Fig. 3). In the front view to the testing machine, the median line is set as Y-axis and the right direction is set as X-axis.
3. Measurement of kinematics
  i) Digitizer
    The ankle joint kinematics was determined by digitizing anatomic landmarks with a 3D digitizing system, the MicroScribe 3DLX (Revware Inc., Raleigh, NC, USA). The accuracy and repeatability of the MicroScribe 3DLX are 0.3 and 0.2 mm, respectively [11].
  ii) Landmark configuration
    For in situ kinematic data collection using Microscribe, landmarks were specified by placing more than 3 points to each independent bone. For digitizing, small crosshead screws were inserted, 3 on the fibula, 6 on the tibia, and 4 on the talus. A set of 3 points on a rigid body can determine a local coordinates system so that 3-Dimensional kinematics of a bone can be traced. And any line connecting sequentially arrayed 2 or more landmarks will give intuitive information on the movement. Three landmarks of the distal fibula (f1, f2, and f3) were specified on the distal anterior fibula, along the superior to inferior direction.
    Six landmarks (t1, t2, t3, t4, t5, and t6) were specified on the distal tibia, alongside distal tibiofibular and talotibial articular surface. Four landmarks (ta1, ta2, ta3, and ta4) were assigned on the talus, alongside the talotibial articulation surface (Fig. 2).
  iii) Joint gaps
    From the landmarks, changes in joint gaps can be measured. The distances are t1-f1, t2-f2, t3-ta1, t4-ta2, t5-ta4, and t6-ta4 are the joint gaps of interest.
  iv) Movements of articulation lines
    The articulation lines are interpreted with lines connecting same-bone landmarks, i.e. tibial articulation line (t1-t2-t3-t4-t5-t6), fibular articulation line (f1-f2-f3), and talar articulation line (ta1-ta2-ta3-ta4). The kinematics in the lines provides changes in alignments of each bones as well as in alignment between bones.
  v) Measurement of foot plantar pressure
    Pressure measurements were obtained using a TekScan sensor 3000E (F-scan research 7.0, TekScan Inc., Boston, MA) calibrated according to the manufacturer’s guidelines. The measurements were processed with F-scan software version 7.00. Using the ‘center of force’ tool and the ‘peak pressure’ tool, the location was measured for each of the two parameters on the sensor for every valgus deformity in all specimens. The total matrix area of the plantar sensor is 12,125 mm^2^(60 × 21 sensels, 236 mm ×51 mm), resulting in a spatial resolution of 9.6 mm^2^ per sensel. The sensor was gently placed into the foot plantar region.

### Statistical analysis

The primary endpoints were the changes in ankle joint motion and the shifts in plantar COF. The changes amount in the dependent variables: gap, point movements and angular motion, and the shift of plantar COF from the neutral position was presented by median and interquartile range (IQR) and also showed on the boxplots. The significance of the changes of those variables was determined by Wilcoxon’s signed rank test under the null hypothesis that the change amount of each variable equals to zero. To compare the change amount between SS-SMO and IS-SMO, Mann-Whitney U test was performed for each variable. All the *p*-values were corrected by Benjamini-Hochberg correction to control the false positive error rate. The correlation coefficients were derived from Spearman’s rank correlation to assess whether there is a relationship between the changes of angles of ankle joints. Posthoc power analysis was conducted using the effect size of difference between SS-SMO and IS-SMO groups.

All statistical analyses were performed using SPSS 14.0KO version and R 3.1.3. version freely available on the web (http://cran.r-project.org/). Values of *P* < 0.05 were considered statistically significant. However, because many comparisons are based on small sample sizes, results of statistical tests with 0.05 < *p* < 0.1 were also reported as suggestive where they have some tendencies.

## Results

**Fig. 4:**
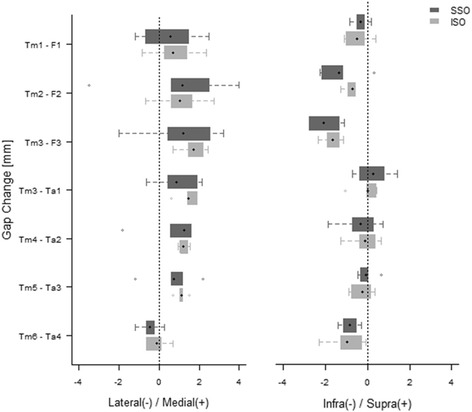
In comparison of the gap change between SS-SMO and IS-SMO distal tibia(Tm), fibula(F), talus(Ta), supra-syndesmotic SMO(SSO), intra-syndesmotic SMO(ISO)

Regarding the changes in gap, a direction of the gap change means a change of the proximal to distal portion. The SS-SMO group showed no significant changes in neigher of the gaps. The IS-SMO group showed significant changes in the gap of Tm3-Ta1, Tm4-Ta2, and Tm5-Ta3 in the medial direction. The changes in the various gaps showed no statistically differences between the two groups (Wilcoxon test, *P* > 0.1). However, large deviation was found in the gap change of Tm4-Ta2 in the medial direction (Table 1) (Fig. 4).

**Table 1:** Change of gap of ankle joint for each supramalleolar osteotomy

Category		SSO		ISO		*P*-value	Posthoc power (%)
		Median	90% C.I.	Median	90% C.I.		
Lateral(−) / Medial (+)	Tm1 - F1	0.66	(−1.19, 2.50)	0.77	(−0.82, 2.37)	1	3.2
	Tm2 - F2	1.54	(−3.48, 3.98)	1.03	(−0.69, 2.75)	1	4.9
	Tm3 - F3	1.48	(−1.98, 3.22)	1.81*	(0.71, 2.46)	1	4.9
	Tm3 - Ta1	0.75	(−0.64, 2.14)	1.52*	(0.60, 1.90)	1	24.5
	Tm4 - Ta2	1.33	(−1.82, 4.47)	1.26*	(0.96, 1.56)	1	2.8
	Tm5 - Ta3	0.87	(−1.20, 2.17)	1.10*	(0.69, 1.52)	1	5.2
	Tm6 - Ta4	−0.44	(−1.19, 0.27)	−0.16	(−0.62, 0.70)	1	10.9
Infra(−) / Supra(+)	Tm1 - F1	−0.34	(−0.85, 0.18)	−0.60	(−1.08, 0.38)	0.98	11.2
	Tm2 - F2	−1.47	(−2.24, 0.33)	−0.76	(−1.26, 0.02)	0.98	24.1
	Tm3 - F3	−2.04	(−2.77, −1.11)	−1.64	(−2.32, −1.13)	0.98	16.7
	Tm3 - Ta1	0.26	(−0.73, 1.41)	0.19	(−1.06, 0.46)	0.98	8.7
	Tm4 - Ta2	−0.20	(−1.85, 0.76)	−0.01	(−1.26, 0.64)	0.98	4.8
	Tm5 - Ta3	−0.16	(−0.47, 0.65)	−0.26	(−0.75, 0.13)	0.98	5.4
	Tm6 - Ta4	−0.83	(−1.38, −0.27)	−0.87	(−2.29, −0.09)	1	3.0

*P*-values were calculated by Wilcoxon rank sum test and adjusted by Benjamini-Hochberg correction

*significant compared to no change, *p* < 0.1 after Benjamini-Hochberg correction

**Fig. 5:**
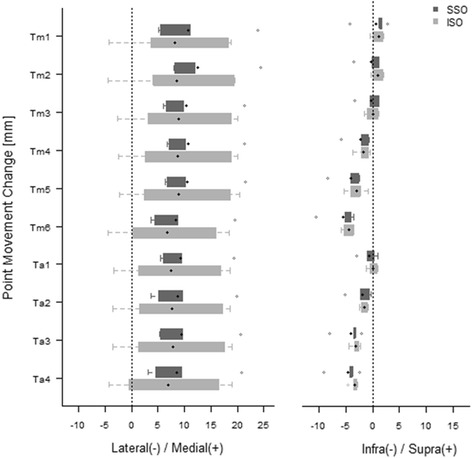
In comparison of the change of point movement between SS-SMO and IS-SMO, distal tibia(Tm), fibula(F), talus(Ta), supra-syndesmotic SMO(SSO), intra-syndesmotic SMO(ISO)

Regarding the changes in point movement, the SS-SMO group showed significant changes in movement in the medial direction for all points, with several points moving in the distal direction. The points in the IS-SMO group also showed points moving in the medial direction, with several points moving in the distal direction. However, the changes in the various point movements showed no statistically significant differences between the two groups (*P* > 0.1) (Table 2) (Fig. 5).

**Table 2:** Change of point movements of ankle joint for each supramalleolar osteotomy

Category		SSO		ISO		*P*-value	Posthoc power (%)
		Median	90% C.I.	Median	90% C.I.		
Lateral(−) / Medial (+)	Tm1	8.28*	(5.03, 23.90)	7.33	(−4.17, 18.83)	0.52	3.7
	Tm2	10.21*	(8.00, 24.40)	7.55	(−4.47, 19.57)	0.52	6.7
	Tm3	8.27*	(6.02, 21.31)	8.69	(−2.71, 20.09)	0.52	3.0
	Tm4	8.66*	(6.74, 21.27)	8.82	(−2.39, 20.03)	0.52	2.7
	Tm5	8.45*	(6.45, 21.52)	9.11	(−2.28, 20.5)	0.52	3.3
	Tm6	6.55*	(3.63, 19.52)	7.04	(−4.34, 18.42)	0.52	3.0
	Ta1	7.66*	(5.42, 19.42)	7.68	(−3.31, 18.67)	0.52	2.5
	Ta2	7.37*	(3.63, 19.80)	7.55	(−3.48, 18.59)	0.52	2.7
	Ta3	7.56*	(5.30, 20.63)	7.76	(−3.46, 18.98)	0.52	2.8
	Ta4	6.99*	(3.21, 20.71)	7.42	(−4.19, 19.03)	0.55	2.9
Infra(−) / Supra(+)	Tm1	1.37	(−4.20, 2.75)	1.01	(−0.49, 2)	0.93	4.4
	Tm2	0.31	(−3.55, 1.19)	0.92	(−0.35, 2.06)	0.93	8.5
	Tm3	−0.17	(−3.32, 1.23)	0.02	(−1.59, 1.12)	0.93	3.8
	Tm4	−1.95*	(−5.92, −0.64)	−1.44*	(−3.75, −0.44)	0.93	6.2
	Tm5	−3.35*	(−8.32, −2.43)	−3.05*	(−5.28, −0.82)	0.93	4.0
	Tm6	−4.58*	(−10.51, −3.61)	−4.46*	(−5.88, −3.45)	0.93	3.0
	Ta1	−0.38	(−1.56, 0.39)	0.09	(−1.21, 1.03)	0.93	12.3
	Ta2	−1.60*	(−5.22, −0.23)	−1.60*	(−2.48, −0.86)	1	2.5
	Ta3	−3.40*	(−8.06, −2.11)	−3.00*	(−4.4, −2.25)	0.93	5.1
	Ta4	−4.03*	(−9.13, −2.47)	−3.26*	(−4.6, −2.84)	0.93	8.1

*P*-values were calculated by Wilcoxon rank sum test and adjusted by Benjamini-Hochberg correction

*significant compared to no change, p < 0.1 after Benjamini-Hochberg correction

**Fig. 6:**
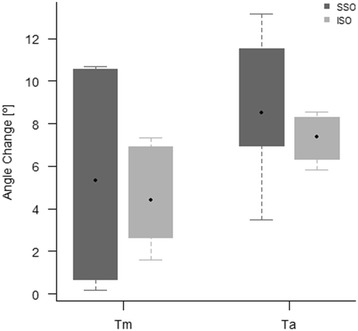
In comparison of the angle change of tibia plafond and talar dome between SS-SMO and IS-SMO, distal tibia(Tm), fibula(F), talus(Ta), supra-syndesmotic SMO(SSO), intra-syndesmotic SMO(ISO), center of force (COF)

The changes in the tibial plafond and talar dome angles, the SS-SMO group showed slightly more changes in the angles on both the tibial plafond and talar dome as compared with the IS-SMO group, in which the changes in the angles were statistically significant in the inferomedial direction(*P* < 0.1). The changes in the angles showed no statistically significant difference between the two groups (*P* > 0.1). The change in the angles of the talar dome showed significantly larger angle than those in tibial plafond in both the groups (Table 3) (Fig. 6).

**Table 3:** Change of angles of ankle joint for each supramalleolar osteotomy

Category		SSO			ISO			*P*-value^a^	Posthoc power (%)
		Median	90% C.I		Median	90% C.I.			
Lateral(−) / Medial (+)	Tm	5.43*	(0.18, 10.67)		4.47*	(1.58, 7.35)		1	6.0
	Ta	8.33*	(3.49, 13.17)		7.32*	(5.85, 8.54)		1	7.3
	Correlation^b^	0.6			0.9**				

*significant compared to no change, *p* < 0.1 after Benjamini-Hochberg correction

**significant correlation, p < 0.1

^a^ *P*-values were calculated by Wilcoxon rank sum test and adjusted by Benjamini-Hochberg correction

^b^Correlation coefficients were calculated by Spearman’s method

**Fig. 7:**
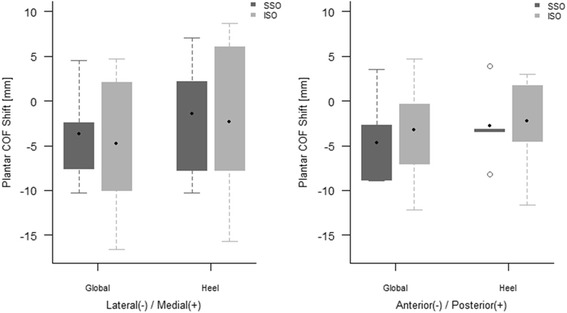
In comparison of the shift of plantar center of force between SS-SMO and IS-SMO, distal tibia-Tm, fibula-F, talus-Ta, SSO(supra-syndesmotic SMO), ISO(intra-syndesmotic SMO)

Regarding the shift of plantar COF, both groups showed no statistically significant shift (*P* > 0.1), were noted in the anterolateral direction. The shift of plantar COF showed no statistically significant difference between groups (*P* > 0.1) (Table 4) (Fig. 7). As a result of posthoc power analysis, this study was underpowered (ranged from 2.5 to 24.1%) to detect the significant differences of all the values between SS-SMO and IS-SMO group due to small sample size.

**Table 4:** Change of plantar COF for each supramalleolar osteotomy

Category		SSO			ISO	*P*-value	Posthoc power (%)
		Median	90% C.I.	Median	90% C.I.		
Lateral(−) / Medial (+)	Global	−2.88	(−10.24, 4.49)	−4.04	(−16.60, 4.68)	1	4.1
	Heel	−1.64	(−10.29, 7.01)	−3.05	(−15.71, 8.62)	1	4.3
Anterior(−) / Posterior(+)	Global	−5.75	(−8.89, 3.54)	−3.71	(−12.17, 4.66)	1	7.1
	Heel	−3.26	(−8.20, 3.85)	−1.38	(−11.60, 2.99)	1	7.3

P-values were calculated by Wilcoxon rank sum test and adjusted by Benjamini-Hochberg correction

*COF* center of force

## Discussion

In our study, SS-SMO and IS-SMO with intact fibula showed changes of movement in gaps the proximal portion to distal portion in the medial and the angular changes in inferomedial direction. The point movements were observed movement in a medial direction as the weight bearing axis on ankle joint was translated. The distal tibia fragment probably showed the shift in the range of syndesmotic clearance because of the weakness of the syndesmosis and instability after SMO. Lee et al. reported that medial ankle osteoarthritis with mortise widening should have performed restoration of width and shape of ankle mortise as well as lateral shifting of weight-bearing axis. He recommended IS-SMO with intact fibula than plafondplasty of intraarticular osteotomy [12]. Lee et al. showed that the incidence of lateral cortical fracture in medial open wedge SMO was less likely to occur at the proximal one-third of the intrasyndesmosis than at the suprasyndesmosis [10]. Knupp et al. reported that with increasing valgus tilt contact area decreased on the anteromedial aspect of the ankle joint [6]. Thus, the medially increased gap in the ankle joint changes the contact area associated with the joint.

Lee et al. reported that after performing SS-SMO with fibula osteotomy, the immediate postoperative radiographs showed that the narrowest area of the joint had shifted from the medial gutter to the tibial plafond-talus area [8]. This can be a result of lateral translation and valgus angulation of the ankle joint [8]. We observed more angle changes in the ankle joint and more translation of axis in case of SS-SMO without statistical significance. The shift of plantar COF in the two groups showed a tendency in the anterolateral direction. The hindfoot was changed in valgus when performed SMO. On the other hand, several biomechanical studies have demonstrated effective correction of malalignment of the ankle joint by the redistribution of ankle joint pressure by using calcaneal osteotomy with either neutral hind foot alignment or valgus deformity [13, 14]. All these studies reported small changes in the ankle joint stresses. This was thought to be due to intact subtalar joint motion in neutral and valgus hindfoot alignment compensating for the altered position of the ground contact point [15].

Our results demonstrate that an oblique supramalleolar opening wedge osteotomy without fibular osteotomy can be used as alternate technique for the treatment of osteoarthrosis of the ankle with neutral alignment and varus deformity. But Bennett et al. recommended concurrent fibular osteotomy to attain derotation and to avoid changing the biomechanics of the ankle joint when rotational osteotomy of the distal tibia is performed [16]. They also observed that the medial malleolus would rotate against the fixed lateral malleolus if distal transverse osteotomy of the fibulais not perfomed [16]. However, Banks et al. and Ryan et al. achieved complete derotation without concurrent fibular osteotomy [17, 18]. A intact fibula also provides additional support for the osteotomized tibia and protects it from sagittal plane angulation. This operation is easily performed, fast, stable, reproducible, and safe for the treatment of osteoarthrosis of the ankle [19].

As per the technique used by M. Knupp [5, 6], after performing SMO, we found there was more translation medially and angulation inferomedially the talar dome than tibia plafond and anterolateralization shift in plantar COF, along with changes in plantar pressure. M. Knupp et al. performed at biomechanical study to determine the effect of supramalleolar varus and valgus alignment on the ankle joint [5]. They evaluated the tibiotalar contact area, tibiotalar force, mean pressure, and peak pressure of tibiotalar in the varus and valgus (5°,10°,and 15°) positions. They reported a mean decrease in the contact area and tibiotalar force transmission in the valgus position and, a mean increase in the contact area and tibiotalar force transmission in the varus position [5]. Schmid et al. evaluated the ankle joint pressure in SMO and lateral calcaneal osteotomy [7]. They reported that both procedures had a significant effect on COF lateralization and peak pressure reduction of ankle joint implies a change from an incongruent joint to a congruent joint [7].

The strength of this study is that the changes in ankle joint motion and plantar pressure after SMO with intact fibula were appropriately measured using Instron modeland TekScan sensor 3000E. This study has a few limitations. First, our cadaver model did not incorporate fibular osteotomy. Stufkens et al. reported in their biomechanical study that when they compared load transfer between procedures that included fibular osteotomy and those that did not, changes in the distal fibula played an important role in determining the contact area and pressure distribution at the ankle [6]. Second, we did not evaluate the COF and mean pressure on the ankle joint. Knupp et al. suggested that in congruent joints, such as, joints with maintained interosseous ligament complex and collateral ligaments, an isolated correction of the distal tibial joint surface angle may not re-establish a physiological load pattern in the ankle joint [5]. Third, although the IS-SMO was shown to be stable because of the inferior tibiofibula ligament, anterior inferior tibiofibula ligament, and posterior inferior tibiofibula ligament, the effect of injury on the syndesmosis after IS-SMO was not studied. Four, The cadaver studies were done on normal joints. The pathologic varus arthritic ankle joint may behave differently. Deltoid release may need to be done to effectively transfer load.

## Conclusions

SS-SMO and IS-SMO with intact fibula showed similar biomechanical effect on the ankle joint. IS-SMO should be considered carefully for the treatment of osteoarthrosis when fibular osteotomy is not performed because lateral cortex fracture is less likely using the IS-SMO.

## Abbreviations

COF: Center of force
IS SMO: Intra-syndesmotic supramalleolar osteotomy
SMO: Supramalleolar osteotomy
SS SMO: Supra-syndesmotic supramalleolar osteotomy
